# Factors influencing the time between onset of illness and specimen collection in the diagnosis of non-pregnancy associated listeriosis in England and Wales

**DOI:** 10.1186/s12879-016-1638-4

**Published:** 2016-06-24

**Authors:** Adedoyin Awofisayo-Okuyelu, Neville Q. Verlander, Corinne Amar, Richard Elson, Kathie Grant, John Harris

**Affiliations:** Gastrointestinal Infections Department, National Infection Service, Public Health England, 61 Colindale Avenue, London, NW9 5EQ UK; National Institute for Health Research, Health Protection Research Unit in Gastrointestinal Infections, Colindale, London, UK; Department of Statistics, Public Health England, 61 Colindale Avenue, London, NW9 5EQ UK; Gastrointestinal Bacterial Reference Unit, Public Health England, 61 Colindale Avenue, London, NW9 5EQ UK

**Keywords:** *Listeria monocytogenes*, Listeriosis, Bacterial Infections, Foodborne diseases

## Abstract

**Background:**

Listeriosis is an opportunistic bacterial infection caused by *Listeria monocytogenes* and predominantly affects people who are immunocompromised. Due to its severity and the population at risk, prompt clinical diagnosis and treatment of listeriosis is essential. A major step to making a clinical diagnosis is the collection of the appropriate specimen(s) for testing. This study explores factors that may influence the time between onset of illness and collection of specimen in order to inform clinical policy and develop necessary interventions.

**Methods:**

Enhanced surveillance data on non-pregnancy associated listeriosis in England and Wales between 2004 and 2013 were collected and analysed. The difference in days between onset of symptoms and collection of specimen was calculated and factors influencing the time difference were identified using a gamma regression model.

**Results:**

The median number of days between onset of symptoms and collection of specimen was two days with 27.1 % of cases reporting one day between onset of symptoms and collection of specimen and 18.8 % of cases reporting more than seven days before collection of specimen. The median number of days between onset of symptoms and collection of specimen was shorter for cases infected with *Listeria monocytogenes* serogroup 1/2b (one day) and cases with an underlying condition (one day) compared with cases infected with serotype 4 (two days) and cases without underlying conditions (two days).

**Conclusions:**

Our study has shown that *Listeria monocytogenes* serotype and the presence of an underlying condition may influence the time between onset of symptoms and collection of specimen.

**Electronic supplementary material:**

The online version of this article (doi:10.1186/s12879-016-1638-4) contains supplementary material, which is available to authorized users.

## Background

Listeriosis is an infection caused by the bacterium *Listeria monocytogenes*. It is a rather uncommon disease but often very severe and with a high case fatality rate [1]. A sub-set of the population including pregnant women and their unborn or new-born babies, the elderly and people who are immunocompromised either as a result of an underlying medical condition or medication are predisposed to listeriosis.

Suspecting a patient has listeriosis can be challenging as infected patients can present with a range of clinical symptoms from non-specific gastroenteritis or influenza-like to those of severe invasive systemic illness. Thus symptoms may include nausea, vomiting, abdominal cramps and diarrhoea, fever, myalgia, general malaise, arthralgia, confusion, neck stiffness and headache. Listeriosis in a healthy adult may present as self-limiting febrile diarrhoea [1]. Where there is central nervous system (CNS) invasion, there is no sign or symptom that securely differentiates listeriosis from other causes of CNS infection [2].

Diagnosis of listeriosis is through the detection of *Listeria monocytogenes* from an otherwise sterile site such as the blood, cerebrospinal fluid, or other sites such as pleural fluid or placenta. Surveillance and public health interventions are important to identify possible sources of contamination and reduce the burden of disease. Effective surveillance of listeriosis relies on the early notification or reporting of a laboratory confirmed case.

The pathway from the initial exposure of a case to the notification of a laboratory confirmation (the ‘exposure to notification’ pathway) is made up of a number of events some of which include the onset of symptoms of listeriosis and the collection of specimen for laboratory testing. There is currently no information available in the literature on the observed time between onset of listeriosis and collection of a specimen, however measuring the duration between each event from exposure to notification can help focus interventions to the appropriate area to ensure timely surveillance and epidemiological investigations. In this study, we review enhanced surveillance data collected over ten years to measure the time difference between onset of listeriosis and the collection of a specimen from a non-pregnancy associated case in England and Wales. We also investigate the factors that may influence this time difference in order to inform clinical policy, guidelines and interventions.

## Methods

Laboratory confirmed cases of non-pregnancy associated listeriosis in England and Wales are reported voluntarily to the Public Health England Centre for Infectious Disease Surveillance and Control (PHE, CIDSC). Two standardised questionnaires, clinical and food history, are completed for each reported case. The clinical questionnaire [3], which is completed by the reporting laboratory, collects specific clinical information including: date of onset, date of specimen collection, presenting symptoms, principal illness, underlying medical conditions (any other ongoing illness, either acute or chronic, reported by the microbiologist including cancer, diabetes, Acquired Immune Deficiency Syndrome (AIDS), cardiovascular disease, liver or kidney disease amongst others), current medications (as some may result in the patent being immunocompromised) and patient survival. The food history questionnaire [4], which is completed by the patient or a proxy, collects basic demographic information including age, ethnicity, gender and postcode of residence. It also collects the patient reported date of onset. Information from both sources are merged, de-duplicated and stored in a database.

In the United Kingdom, deprivation scores can be derived from the postcode of the area of residence. Postcodes were used to map geographical areas with an average population size of 1,500 people for which a deprivation score is then calculated using the Index of Multiple Deprivation (IMD) 2007. The IMD 2007 is a composite measure based on 38 indicators grouped in seven domains: income; employment; health deprivation and disability; education, skills and training; barriers to housing and services; crime; living environment [5]. The calculated deprivation score is then assigned to a corresponding postcode and this is ranked and then divided into quintiles with 1 being the least deprived and 5 the most deprived.

Date of onset of symptom, for the purpose of this study, is defined as the first day the patient developed symptoms of listeriosis. This is collected using both the clinical and food history questionnaires and checked to ensure consistency. Where there is a difference in the recorded dates, the date reported on the food history questionnaire is used as this is reported by the patient or a close relative. The date of specimen is defined as the reported date when the first clinical specimen was collected from a suspected case of listeriosis.

The time between onset of symptoms and collection of specimen was calculated as the difference in days between the date of symptom onset and the date of specimen collection. Where either or both dates were unknown, or when the date of specimen was before the date of symptom onset, cases were excluded from this study.

### Case definition

A case of non-pregnancy associated listeriosis is confirmed when *L.monocytogenes* has been isolated from a sterile site such as the blood or cerebrospinal fluid or other site. Infants over 28 days of age were included in the case definition as they are not regarded as pregnancy associated cases.

### Microbiological methods

Serogrouping was performed following the multiplex PCR (polymerase chain reaction) developed by Doumith and colleagues [6].

### Statistical analysis

We examined the patients’ characteristics as functions of the time to collection of specimen using a generalised linear regression model, accounting for the positively skewed data by assuming the errors are gamma distributed and using an identity link function. The time difference between the reference category and the group(s) of interest in each variable was estimated by using a multivariable model consisting of all the explanatory variables together.

A likelihood ratio test (LRT) was used to compare the fit of the model which included all the variables and the alternative model which excluded the variable of interest. The resulting *p*-value indicated whether the more complex model had a significantly better fit than the simpler one and, if so, then the variable of interest significantly improved the fit. We have reported the *p*-values of the likelihood ratio tests rather than the individual *p*-values of the regression analysis to show how the presence or absence of each variable in the model affects the outcome. Irrespective of wide confidence intervals or confidence intervals that include zero, a significant p-value indicates how the variable affects the time to collection of specimen as judged by the improvement in model fit. Wide confidence intervals mean that the effects are imprecisely estimated.

A chi-square test for association was used to check for correlations between the serogroups and deprivation quintiles. We confirmed that fewer than 20 % of the cells in the five by four contingency table had an expected value less than five.

The significance level was chosen to be 5 %. Statistical analyses were carried out using Stata version 12.1.

## Results

### Study population

A total of 1608 non-pregnancy associated cases of listeriosis in England and Wales were reported to the enhanced surveillance system during the study period, between January 2004 and December 2013. Of these, 23.1 % (372/1608) did not have either date of specimen or date of onset recorded. A further 1.6 % (26/1608) of the cases had the date of specimen before the date of onset. These records were excluded from further analysis leaving 1210 cases (75.2 %) for analysis.

### Exploration of factors associated with time between onset of illness and specimen collection

The median number of days between onset of symptom and collection of specimen was two days with a range of 0–81 days. Seventy per cent of the study population had a specimen collection within three days of onset and 18.8 % had a specimen collection after seven days of onset of illness (Table 1). Collection of specimen occurred 60 days or more after date of onset for six cases (Table 2).

**Table 1:** Cases of non-pregnancy associated listeriosis in England and Wales

Variable	2004–2013
Total number of cases	1608
Number of cases without date of onset or date of specimen	372 (23.1 %)
Number of cases with date of specimen before date of onset	26 (1.6 %)
Number of cases with date of specimen equal to date of onset	368 (22.9 %)
Number of cases reporting one or more days before collection of specimen	842 (52.4 %)
1 day	228 (27.1 %)
2–3 days	252 (29.9 %)
4–5 days	126 (14.9 %)
6–7 days	78 (9.3 %)
More than 7 days	158 (18.8 %)

**Table 2:** Cases reporting over 60 days between onset of illness and collection of specimen

Number of days before collection of specimen	Age group	Serogroup	Principal illness	Comments	Outcome
62 days	60+	1/2a	Other illness	Previous aortic valve replacement	Died
64 days	60+	4	Septicaemia	Enlarged prostate gland with complains of backache and abdominal pain	Alive
69 days	60+	4	Other illness	Presence of underlying condition not known	Alive
73 days	<60	unknown	Meningitis	HIV	Alive
76 days	60+	4	Septicaemia	No underlying condition	Alive
81 days	<60	4	Gastroenteritis	Ulcerative colitis and on omeprazole	Alive

The number of cases of listeriosis in each age group increased with age. Of the seven cases between 0 and 9 years of age, two were infants less than 12 months old (three and eight months) and three were under five years of age (two and three years old) and the remaining two were five years old. With the exception of cases aged over 70 years old that had a median of one day before collection of specimen, the median days between onset of symptom and collection of specimen for the rest of the cases was two days (Table 3).

**Table 3:** Characteristics of non-pregnancy associated cases of listeriosis influencing time between onset of illness and collection of specimen

Patient characteristics	Number of cases^a^(%) (*N* = 1210)	Median number of days between onset of symptoms and collection of specimen (IQR) (in days)	Change in time difference between onset of symptoms and collection of specimen			
			Unadjusted (95 % C1) (in days)	*P*-value of LRT^b^	Adjusted (95 % CI) (in days) No of observations - 886	*P*-value of LRT^b^
Age						
0–9 years	7 (0.6)	2 (1 to 18)	0	0.18	0	0.08
10–29 years	32 (2.6)	2 (0 to 5)	−2.5 (−11.6 to 6.5)		−3.1 (−15.3 to 9.2)	
30–49 years	103 (8.5)	2 (0 to 5)	−1.8 (−10.7 to 7.1)		−4.5 (−16.6 to 7.5)	
50–69 years	448 (37.0)	2 (0 to 5)	−2.9 (−11.7 to 5.8)		−4.4 (−16.3 to 7.5)	
70+ years	620 (51.2)	1 (0 to 4)	−2.5 (−11.2 to 6.2)		−3.6 (−15.6 to 8.2)	
Gender						
Females	537 (44.4)	2 (0 to 4)	0	0.77	0	0.27
Males	673 (55.6)	1 (0 to 4)	+0.1 (−0.8 to 0.9)		+0.3 (−0.5 to 1.2)	
Ethnicity						
White British	1057 (87.4)	1 (0 to 4)	0	0.24	0	0.11
Others	153 (12.6)	2 (1 to 5)	+0.5 (−0.9 to 1.9)		+0.8 (−0.6 to 2.3)	
Deprivation						
1 (least)	224 (18.6)	1.5 (0 to 4)	0	0.05	0	0.02
2	235 (19.5)	2 (1 to 4)	+0.2 (−0.9 to 1.5)		−0.1 (−1.2 to 1.0)	
3	229 (19.1)	2 (0 to 5)	+1.1 (−0.3 to 2.4)		+1.2 (−0.2 to 2.7)	
4	260 (21.6)	1 (0 to 4)	+1.1 (−0.2 to 2.5)		+1.0 (−0.3 to 2.3)	
5 (most)	255 (21.2)	2 (0 to 5)	+0.7 (−0.5 to 2.0)		+0.7 (−0.5 to 2.0)	
Unknown	7					
Serogroup						
1/2a	341 (32.8)	1 (0 to 4)	0	<0.001	0	<0.003
1/2b	97 (9.3)	1 (0 to 3)	−1.3 (−2.7 to −0.6)		−1.5 (−2.7 to −0.3)	
1/2c	36 (3.5)	1 (0 to 4.5)	−0.9 (−3.0 to 1.2)		−0.6 (−2.5 to 1.2)	
4	566 (54.4)	2 (0 to 5)	+0.6 (−0.4 to 1.6)		+0.2 (−0.7 to 1.2)	
Unknown	170					
Underlying condition						
Absent	168 (15.1)	2 (1 to 5)	0	<0.002	0	0.001
Present	942 (84.9)	1 (0 to 4)	−1.3 (−2.8 to 0.2)		−1.5 (−3.1 to −0.03)	
Unknown	100					
Presenting illness						
Other illness	91 (8.1)	2 (0 to 6)	0	<0.001	0	<0.001
Gastroenteritis	46 (4.1)	1 (0 to 6)	+0.8 (−2.8 to 4.5)		+.2.7 (−1.5 to 7.1)	
Meningitis	140 (12.4)	2 (1 to 5)	−0.8 (−3.1 to 1.6)		−0.7 (−3.1 to 1.6)	
Septicaemia	618 (54.8)	1 (0 to 4)	−1.5 (−3.6 to 0.4)		−0.8 (−2.8 to 1.2)	
Septicaemia and Gastroenteritis	83 (7.4)	2 (0 to 6)	+0.1 (−2.7 to 2.9)		+0.4 (−2.2 to 3.2)	
Meningitis and Septicaemia	143 (12.7)	2 (0 to 3)	−2.4 (−4.5 to −0.2)		−1.7 (−3.8 to 0.4)	
Meningitis and Septicaemia and Gastroenteritis	7 (0.6)	6 (4 to 10)	+1.2 (−7.4 to 9.9)		+2.2 (−6.5 to 11.1)	
	82					
Survival						
Alive	768 (66.7)	2 (0 to 4)	0	0.52	0	0.76
Died	383 (33.3)	1 (0 to 4)	−0.2 (−1.2 to 0.8)		+0.1 (−0.8 to 1.0)	
Unknown	59					

^a^Data available as Additional file 1: Data of non-pregnancy associated listeriosis

^b^Likelihood ratio test

In our study population, the male to female ratio was 1.2: 1, and white British cases accounted for 87.4 %, however, there was no significant difference in the time between onset and collection of specimen in either gender as well as the different ethnic groups (White British and non-White British).

Ninety-nine per cent (1203/1210) of cases had their postcode of residence reported and 21.2 % lived in the most deprived areas. Although there is no significant difference in the time period between onset of illness and collection of specimen for the different deprivation quintiles, there is some association between deprivation and the time between onset of illness and collection of specimen as judged by the improvement in fit with increased duration for the third and fourth quintiles (Table 3). We did not observe any correlation between the deprivation quintiles and the different serogroups (*p*-value for chi-square test = 0.6).

Serogrouping was carried out on *L. monocytogenes* isolated from the 1040 cases (85.9 %) referred to the reference laboratory and more than half of these were of serogroup 4. Compared with cases infected with serogroup ½ a, specimens were collected 1.3 days earlier for cases infected with serogroup 1/2b (95 % CI −2.7 days to −0.6 days), and this difference was still significant after other factors were accounted for in the regression model (Table 3).

The presence or absence of an underlying medical condition before the onset of listeriosis was recorded for 91.7 % of the cases (1110/1210). Eighty-four per cent of these reported having an underlying medical condition and had specimen collected 1.5 days earlier (95 % CI −3.1 days to −0.03 days) compared with cases without an underlying condition (Table 3).

The reported presenting illness were septicaemia (54.8 %; 618/1128), meningitis (12.4 %; 140/1128), and gastroenteritis (4.1 % (46/1128). Some cases reported a combination of these illnesses while others reported other illnesses including pneumonia and endocarditis. Over half of the cases with an underlying condition presented with septicaemia while about 20 % of cases without an underlying condition presented with meningitis (results not shown). Of the cases infected with serogroup 4, 51.4 % presented with septicaemia, 16.0 % presented with meningitis, 15.2 % presented with both meningitis and septicaemia and 2.9 % presented with gastroenteritis. Of the cases infected with serogroup 1/2b, 53.9 % presented with septicaemia, 12.1 % presented with both meningitis and septicaemia, 10.9 % presented with meningitis and 2.2 % presented with gastroenteritis (Table 4). The median number of days between onset of illness and collection of specimen was longest for cases presenting with a combination of all three illnesses (six days), although this was not statistically different from the rest of the cases (Table 3).

**Table 4:** Proportion of presenting illness by infecting serogroup

Serogroup (N)	Other illness (N)	Gastroenteritis (N)	Meningitis (N)	Septicaemia (N)	Septicaemia and Gastroenteritis (N)	Meningitis and Septicaemia (N)	Meningitis and Septicaemia and Gastroenteritis (N)	Unknown
1/2a	8.0 (26)	6.2 (20)	8.3 (27)	57.4 (186)	8.6 (28)	10.8 (35)	0.6 (2)	17
1/2b	10.9 (10)	2.2 (2)	10.9 (10)	53.9 (49)	9.9 (9)	12.1 (11)	0	6
1/2c	14.7 (5)	0	8.8 (3)	58.8 (20)	2.9 (1)	11.7 (4)	2.9 (1)	2
4	7.1 (37)	2.9 (15)	16.0 (84)	51.4 (270)	6.9 (36)	15.2 (80)	0.6 (3)	41
Unknown	13	9	16	93	9	13	1	16

The case fatality rate of the study population was 33.3 %, however, the number of days between onset of illness and collection of specimen did not influence the outcome of listeriosis i.e., whether a case survived or died.

A sensitivity analysis was undertaken by using ordinal logistic regression in place of gamma regression but this yielded comparable results to those presented. In both approaches, we analysed age first as a continuous variable and then a categorical variable. The results were also comparable.

## Discussion

Following exposure to *L. monocytogenes*, there are certain necessary events that determine the time period between exposure and implementation of epidemiological interventions. This time period, for the purpose of this study has been called the ‘exposure to intervention’ pathway. The events in the pathway after exposure are: onset of illness, contact with medical care, collection of specimen, isolation of *L. monocytogenes* and notification of a laboratory confirmation. Although treatment can be initiated at any point in the pathway, public health interventions can only commence after notification. The latter can be further delayed when additional molecular typing has to be carried out. A delay between any of the events on the pathway could result in delays in the notification of confirmed cases of listeriosis which can have implications on the effectiveness of public health interventions [7] such as the identification of potential sources of contamination, and the prevention of further cases.

Estimating the incubation period of gastrointestinal pathogens, which is the time between consumption of a contaminated food item (exposure) and onset of illness, can be difficult [8], and even more challenging for listeriosis due to its non-specific clinical symptoms. The incubation period of listeriosis can be less than 24 h and as long as 90 days [9]. Cases involving the CNS have incubations periods ranging from 1 – 14 days and cases of septicaemia have incubation periods between one and twelve days [8]. Symptoms of gastroenteritis can develop from 24 h following infection [10, 11].

This study calculated the median time difference between onset of symptoms and collection of specimen and we have presented results showing that cases with an underlying condition and cases infected with *L. monocytogenes* serogroup 1/2b had shorter time periods compared with other cases of listeriosis.

The presence of an underlying condition can either decrease or increase the time period between onset of illness and specimen collection, as persons with underlying conditions may have frequent access to health care and the possibility of early specimen collection, or conversely, the presence of an underlying condition in some may make diagnosing listeriosis difficult due to its non-specific symptoms. In the study population described here, the time period for cases with underlying conditions was shorter. In England and Wales, about 85 % of non-pregnancy associated cases report underlying conditions and certain conditions result in higher risk of listeriosis compared to others [12]. Frequently reported conditions were malignancies, diseases of the circulatory and digestive system. Cases with these conditions are likely to be hospital in-patients or have frequent contact with health services thereby increasing the probability of an early specimen collection. However, not all cases with underlying conditions will have a short time period between onset of illness and collection of specimen. Some case reports show patients with a time period of three or four days [13, 14]. The presence of an underlying condition may mask the symptoms of listeriosis as the latter can be non-specific. As the diagnosis of listeriosis primarily relies on blood cultures, it can be extremely difficult to suspect when the patient presents with undifferentiated illness.

Similar to other populations [15, 16], *L. monocytogenes* serogroups 4 and 1/2a were the most frequently reported serogroups in our study population, however the time difference between onset of illness and collection of specimen was shorter for cases infected with *L. monocytogenes* serogroup 1/2b. The reason for this time difference is unknown as the distribution of presenting illness is similar across the different serogroups. In this study, the low numbers of cases with serogroup 1/2b means this could be a spurious effect. The virulence difference between *L. monocytogenes* serogroups, where serogroup 1/2a is considered less virulent [17], could result in cases having milder symptoms and therefore presenting to a General Practitioner (GP) later accounting for the longer time difference between onset of symptoms and collection of specimen. It should be noted that the pathogenic potential of *L. monocytogenes* and what makes one strain more virulent than another is poorly understood.

Ethnic and gender differences [18] as well as socio-economic differences contribute to health inequalities particularly in accessing secondary and tertiary health care however; these differences may not be related to the health seeking behaviours of patients. According to a UK study [19]; all patients had at least an equal probability of seeking immediate healthcare following perception of need. This may explain the similarity in the time periods between onset of symptoms and collection of specimen for the cases irrespective of gender, ethnicity and socio-economic status. Furthermore, the severity of listeriosis may remove the choice to seek help causing all cases to seek medical care equally as soon as possible. This shows that a patient’s demographic characteristics otherwise do not influence the time period between onset of symptoms and specimen collection.

There was also no difference in the time between onset of symptoms and collection of specimen for all cases irrespective of the presenting principal illness reported (meningitis, septicaemia or gastroenteritis). The low *p*-values nevertheless indicate that the presenting illness might have an effect on the time difference between onset of symptoms and collection of specimen, however, the direction of effect is unknown.

The date of onset requested on the standardised questionnaires is the first day the patient developed symptoms of listeriosis. However, there may be variations in the interpretation of the question depending on the interviewer. Also, for cases with an underlying condition, it may be difficult to accurately identify when symptoms of listeriosis started. These misinterpretations may have impacted our estimates of the onset of symptoms and thereby resulting in either an overestimation or an underestimation of the time period between onset of illness and collection of specimen particularly in cases that reported over 60 days between onset of illness and collection of specimen.

Pregnancy associated cases were excluded from this study because the non-systematic method of reporting cases could introduce a bias. Both mother and baby (infant under 28 days of age) are regarded as one pregnancy associated case, and details of either mother or baby is recorded in the database. The selection of which case is recorded depends on which laboratory result is received first. If the results of more babies are reported first, the age distribution will be skewed to the 0–9 years, and if the results of more mothers are reported first, the gender distribution will be skewed towards females.

## Conclusion

We have shown here that the infecting *L. monocytogenes* serotype and the presence of an underlying condition can influence the time difference between onset of symptoms and collection of specimen. Physicians, emergency doctors and infectious disease doctors should be made aware of people at-risk of listeriosis, and encouraged to consider listeriosis as a differential diagnosis so that they order tests that hasten diagnosis. Factors influencing time between onset of symptoms and collection of specimen are not only limited to patient characteristics, but could also include health care associated factors. In this study, we have only identified some of the patients’ characteristics that influence the time difference between onset of symptoms and collection of specimen and not the factors associated with health care delivery, hence, further research is needed to identify factors such as health care accessibility and delivery. In addition, the weight of each factor (health care or patient) in influencing the time to collection of specimen also needs to be determined so as to help target health interventions and policies where they are most needed.

## Abbreviations

AIDS, acquired immune deficiency syndrome; CNS, central nervous system; GP, general practitioner; IMD, index of multiple deprivation; LRT, likelihood ratio test; PCR, polymerase chain reaction; PHE, CIDSC, Public Health England Centre for Infectious Disease Surveillance and Control

## Additional file

Additional file 1: Data of non-pregnancy associated a listeriosis. (XLSX 94 kb)
